# Modulation of TRP Channel Activity by Hydroxylation and Its Therapeutic Potential

**DOI:** 10.3390/ph10020035

**Published:** 2017-03-27

**Authors:** Yagnesh Nagarajan, Grigori Y. Rychkov, Daniel J. Peet

**Affiliations:** 1 School of Biological Sciences, University of Adelaide, Adelaide 5005, SA, Australia; yagnesh.nagarajan@adelaide.edu.au; 2 School of Medicine, University of Adelaide, Adelaide 5005, SA, Australia; grigori.rychkov@adelaide.edu.au; 3 South Australian Health and Medical Research Institute (SAHMRI), Adelaide 5005, SA, Australia

**Keywords:** TRPA1, TRPV3, hydroxylation, PHD, FIH, HIF, oxygen, hypoxia

## Abstract

Two transient receptor potential (TRP) channels—TRPA1 and TRPV3—are post-translationally hydroxylated, resulting in oxygen-dependent regulation of channel activity. The enzymes responsible are the HIF prolyl hydroxylases (PHDs) and the asparaginyl hydroxylase factor inhibiting HIF (FIH). The PHDs and FIH are well characterized for their hydroxylation of the hypoxic inducible transcription factors (HIFs), mediating their hypoxic regulation. Consequently, these hydroxylases are currently being targeted therapeutically to modulate HIF activity in anemia, inflammation, and ischemic disease. Modulating the HIFs by targeting these hydroxylases may result in both desirable and undesirable effects on TRP channel activity, depending on the physiological context. For the best outcomes, these hydroxylases could be therapeutically targeted in pathologies where activation of both the HIFs and the relevant TRP channels are predicted to independently achieve positive outcomes, such as wound healing and obesity.

## 1. Introduction

The transient receptor potential (TRP) channels are non-selective cation channels broadly expressed in most tissues in the body. TRP channels play a role as cellular sensors which respond to a diverse range of extracellular and intracellular stimuli, including second messengers, chemicals, temperature, redox state, mechanical stimulation, and osmolality [1,2,3,4]. Recent research suggests that abnormal activity of some members of the TRP super-family contributes to human pathologies such as cancer, diabetes, lung and liver fibrosis, chronic pain, ischemia-reperfusion injury, pulmonary hypertension, irritable bowel disease, drug toxicity, and others [2,5,6,7,8,9].

TRP channels are formed by homo- or hetero-tetramers of TRP proteins, with each a TRP monomer comprised of six transmembrane domains flanked by intracellular N- and C-terminal domains. Similar to voltage-gated channels, a conserved pore-forming loop between transmembrane domains 5 and 6 in TRP proteins is responsible for cation permeability. Although most TRP channels under physiological conditions conduct more Na^+^ than Ca^2+^, the Ca^2+^ permeability of TRP channels is considered to be important for the maintenance of intracellular Ca^2+^ signaling and homeostasis.

Apart from specific stimuli that gate TRP channels, TRP channel activity can be further controlled by a range of post-translational modifications, including phosphorylation and glycosylation (reviewed in [10]). Two specific TRP channels—TRPA1 and TRPV3—are modified post-translationally by hydroxylation (an oxygen-dependent modification), which mediates a characteristic response to hypoxia [11,12]. The hydroxylases responsible for these modifications have emerged as key therapeutic targets for a range of human diseases due to their direct regulation of the hypoxia-inducible factors (HIFs, the central transcription factors that mediate the genomic response to hypoxia), with important therapeutic implications for TRP channels.

## 2. Hydroxylation-Dependent Regulation of Hypoxic Gene Expression

**Figure 1:**
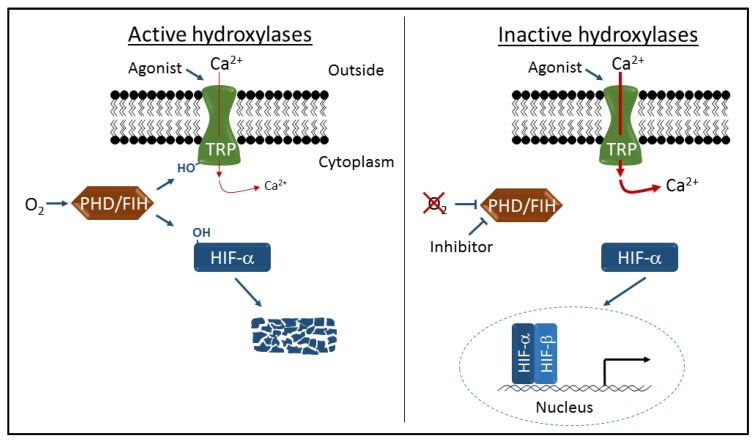
Schematic representation of transient receptor potential (TRP) channel and hypoxia-inducible factor (HIF) regulation by the prolyl hydroxylase containing enzymes (PHD) and asparaginyl hydroxylase factor inhibiting HIF (FIH) hydroxylases. Oxygen-dependent hydroxylation (-OH) of TRPA1 and TRPV3 channels inhibits cation entry through activated channels, and hydroxylation of HIFα proteins leads to proteolytic degradation and transcriptional repression. Inhibition of hydroxylase activity by hypoxia or specific inhibitors leads to increased cation entry and robust HIF-dependent gene activation.

The prolyl hydroxylase containing enzymes (PHDs) and the asparaginyl hydroxylase factor inhibiting HIF (FIH) belong to a conserved family of 2-oxoglutarate-dependent dioxygenases, and act as cellular oxygen sensors [13]. These oxygen-sensing enzymes catalyze the addition of hydroxyl (OH) groups to specific prolyl or asparaginyl residues on target proteins, altering their activity. The three closely related PHD enzymes (PHD1, 2, and 3, also referred to in the literature as EGLN2, 1, and 3) were originally characterized through their oxygen-dependent hydroxylation of two proline residues within the HIFα subunit of the heterodimeric HIF transcription factors. HIFα hydroxylation occurs in normoxia and promotes ubiquitination and rapid proteosomal-mediated degradation (reviewed in [14]). In hypoxia the activity of the PHDs decreases, allowing the HIFα subunits to avoid hydroxylation, ubiquitylation and subsequent degradation, and consequently the stabilized HIFα mediates robust gene induction in response to hypoxia (Figure 1). Thus, the PHDs act as the essential oxygen sensors in this pathway and are the primary regulators of the HIF-driven genomic response to hypoxia. FIH was subsequently characterized as a related hydroxylase that hydroxylates a single asparaginyl residue in a transactivation domain of the HIFα subunit, resulting in transcriptional repression in hypoxia (reviewed in [15]). As with the PHDs, hypoxia results in the loss of efficient oxygen-dependent hydroxylation, and alleviates the transcriptional repression, contributing to increased hypoxic gene induction mediated by the HIFs. However, the role of FIH in modulating oxygen-dependent gene regulation via the HIFs is modest compared to the PHDs, and its physiological importance less well characterized.

The PHDs and FIH also have a number of substrates in addition to the HIF proteins, although the physiological consequence of hydroxylation on these substrates has not been well established. Other substrates for the PHDs include pyruvate kinase M2, RNA polymerase II, erythropoietin receptor, eukaryotic elongation factor 2, and beta(2)-adrenergic receptor [16]. FIH has been shown to hydroxylate a number of proteins containing ankyrin repeat domains (ARDs), although in most cases there is no obvious effect on the function of the hydroxylated substrate [17]. However, two more recently identified ARD substrates and one non-ARD substrate show some hydroxylation-dependent changes in activity [11,18,19].

## 3. Hydroxylation-Dependent Regulation of TRP Channel Activity

TRPA1 is activated by a range of chemical agonists (both endogenous and exogenous), mechanical stimulation, and cold temperature (reviewed in [20]). It has been implicated in a number of pathologies, including acute inflammation and cartilage degeneration in osteoarthritis [21], urinary bladder pain in cystitis [22], neuropathic and inflammatory pain, migraine, and familial episodic pain syndrome [23,24]. Takahashi and colleagues reported that TRPA1 is also sensitive to changes in oxygen, undergoing oxygen-dependent hydroxylation on a single proline residue within the ARD by the PHDs in normoxia [12]. Their data supported hydroxylation-dependent inhibition of channel activity that was rapidly reversed in hypoxia, when the oxygen-sensing PHDs have greatly diminished activity, leading to an increase in activity of the unmodified channel. Furthermore, they demonstrated that TRPA1 channels also respond to oxygen through the oxidation in hyperoxia of specific cysteine residues located within the seventeenth ARD (Cys 633 and Cys 856). A recent study proposes a role for TRPA1 hydroxylation in the hypoxic ventilator response [25]. In addition, this oxygen-dependent hydroxylation has been implicated in mediating the response to cold temperature through the production of reactive oxygen species [26]. Of interest, TRPA1 also responds to ischemia in oligodendrocytes, mediating Ca^2+^ entry and subsequent damage to myelin, although the role of hydroxylation in this response has not been explored [27].

TRPV3 was originally characterized as a warm temperature sensing channel [28,29,30], and is also activated by endogenous and exogenous chemical ligands. It plays roles in the maintenance of epidermal barrier function, hair growth, and nociception, and has been implicated in pathologies associated with dermatitis, pruritus, inflammation, ischemia, and wound healing [31,32]. The TRPV3 channel has also been shown to be hydroxylated, but on a single asparaginyl residue within the ARD, by FIH [11]. In common with the PHD-mediated hydroxylation of TRPA1, FIH-dependent hydroxylation of TRPV3 in normoxia inhibits channel activity, but is rapidly reversed in hypoxia, leading to increased hypoxic TRPV3 activity (Figure 1).

In the case of both TRPA1 and TRPV3, the mechanism by which hydroxylation modulates channel activity has not been determined. For example, hydroxylation may influence the physical structure of the channels and consequently influence gating, or may affect agonist binding, multimerisation with other TRP channels, or the recruitment of accessory proteins that modulate function. In addition, while the evidence for TRPA1 and TRPV3 hydroxylation is strong, as with other non-HIF substrates, the physiological role for these hydroxylation events remains poorly defined.

Whether other TRP channels are regulated by hydroxylation, mediated by the PHDs, FIH, or other hydroxylases remains unclear. In addition to TRPA1 and TRPV3, it has also been reported that peptides from the ARD of TRPV4 can be hydroxylated in vitro by FIH [33]. We have investigated the hydroxylation of nine other TRP channels predicted using bioinformatics to be substrates of FIH (TRPC1, C3, C4, C5, C6, V2, V5, V6), but found no evidence for hydroxylation using in vitro hydroxylation assays (unpublished data). Furthermore, other than TRPA1, none of the nine other TRP channels analyzed by Takahashi et al. displayed hypoxic induction, including TRPV3. However, it is important to note that these experiments were performed in 10% oxygen, which is relatively mild hypoxia and unlikely to influence FIH-mediated hydroxylation [12,34]. A recent report of interest implicated mouse TRPC2 in sensing low oxygen within olfactory epithelium [35]. While it is unclear whether this response involves hydroxylation (and TRPC2 is only a pseudogene in humans), it does support the hypothesis that other TRP channels may also be regulated in a similar manner to TRPA1 and TRPV3. The formation of heterotetramers with other channels may also cause the modification of one channel to influence the activity of another. For example, TRPV3 is known to form functional heterotetramers with TRPV1 [29,36], hence regulation of TRPV3 via hydroxylation may indirectly influence the activity of a TRPV1/V3 heterotetramer where V1 is activated.

Although the body of literature on TRP-hydroxylation is clearly very limited and more research is required to ascertain the physiological relevance, these studies establish that the oxygen-dependent hydroxylation of at least two members of the TRP superfamily confers hypoxic responsiveness to these channels. These findings have important implications regarding the potential for novel therapeutic manipulation of the activity of these specific TRP channels via altered hydroxylation, either directly or as a consequence of the therapeutic targeting of the PHDs and FIH to regulate other substrates.

## 4. Therapeutic Targeting of PHDs and FIH to Activate HIF

Given that hypoxia contributes to the pathophysiology of most major diseases, including myocardial and cerebral ischemia, vascular disease, and cancer, it is not surprising that therapeutic manipulation of the ubiquitous HIF pathway has become a highly sought after goal. The PHDs and FIH are attractive therapeutic targets, as they are well characterized functionally and structurally [37,38,39,40], can be expressed recombinantly [41,42], and inhibition results in HIF activation, with the PHDs of particular interest as the primary regulators of the HIFs [43]. However, specificity is an important issue when targeting PHDs or FIH in human pathologies, given the large family of related 2-oxoglutarate-dependent oxygenases, the hundreds of target genes directly regulated by the HIFs that influence diverse biological processes including erythropoiesis, angiogenesis, metabolism, cell migration and survival, and the other less well characterized substrates of the PHDs and FIH, including the TRP channels.

To date, numerous PHD inhibitors have been developed, with a number in pre-clinical and clinical trials (reviewed in [43]). The initial focus has been on the treatment of anemia, as erythropoietin is a direct target gene of HIF, with two PHD inhibitors currently in Phase 3 clinical trials showing considerable promise. Additional clinical and preclinical trials have also targeted ischemia and inflammation, with other pathologies also under investigation. Specificity for different HIF-mediated outcomes is achieved through the use of inhibitors that display selectivity for one or more of the PHDs (the three different PHDs show some specificity for different HIFα proteins, and consequently regulate distinct target gene responses), as well as distinct delivery and treatment regimes. Little is known regarding the consequence of these treatments on non-HIF targets of the PHDs, including TRPA1.

Preliminary screens have also been performed to identify specific inhibitors of FIH [44,45], but given the modest effects on HIF activity mediated by FIH, it has not been a focus of pharmaceutical research. However, one potential advantage of targeting FIH is that additional specificity may be achieved compared with the PHDs, as FIH only influences the expression of a discrete subset of HIF target genes, and shows some specificity for different HIF isoforms [34,46].

## 5. Therapeutic Targeting of PHDs and FIH to Modulate TRP Channel Activity

Therapeutic manipulation of the PHDs and FIH is likely to modulate the activity of TRPA1 and TRPV3, respectively. Importantly, inhibition of hydroxylase activity—which has been the focus of therapeutic manipulation to achieve HIF activation—has the potential to increase TRPA1 and TRPV3 activity through abrogation of hydroxylation. However, given the key role of the hydroxylases—specifically the PHDs—in regulating the ubiquitously expressed HIFs, specificity is likely to be a major issue. Importantly, while specific inhibitors of the PHDs and FIH should theoretically also modulate TRPA1 and TRPV3 activity, respectively, through altered hydroxylation, this needs to be determined experimentally. This characterization should include inhibitors that show specificity for each of the hydroxylases, including those currently in clinical trials, and their consequence on both hydroxylation and activity. In the case of both TRPA1 and TRPV3, loss of hydroxylation does not appear to independently activate the channels, but rather the limited data are consistent with an increase in the activity of an already active channel. Hence therapeutic targeting of the PHDs or FIH might only modulate the activity of an already active channel. Physiologically, this would depend on the presence of endogenous chemical agonists, mechanical stimuli, or temperature to activate the channels.

The knowledge that TRPA1 is also regulated by PHD-mediated hydroxylation should also inform the current clinical and preclinical trials using PHD inhibitors to activate the HIFs. For example, a number of pre-clinical studies have targeted inflammation in mouse models of colitis with mostly positive outcomes [43]. However, in similar models of colitis, TRPA1 channels are shown to contribute to disease pathology [47], with TRPA1 antagonists being designed and trialed for treatment of inflammation and pain [48]. Consequently, the activation of TRPA1 achieved with PHD inhibitors is likely to promote rather than inhibit disease pathology and associated pain.

Targeting TRPV3 activity via FIH should be more specific, given the modest role for FIH in HIF regulation and the apparent lack of an effect of hydroxylation on the activity of most characterized ARD substrates [17]. However, this is complicated by the metabolic phenotype of FIH null mice, which are viable and display hypermetabolism, hyperventilation, lowered body mass and adiposity, resistance to weight gain when fed a high fat diet, and insulin hypersensitivity. This phenotype is not readily explained by the known roles for FIH in regulating the HIFs or other characterized ARD substrates, and supports the existence of one or more additional substrates involved in controlling metabolism [49].

A more prudent strategy to therapeutically target the PHDs or FIH to modulate TRP channel activity would focus on pathologies where activation of both the HIFs and the relevant TRP channels are predicted to independently achieve positive outcomes. For example, both TRPV3 and HIF have been implicated in wound healing [50,51], hence the therapeutic inhibition of FIH may promote wound healing through two independent pathways, via activation of both HIF and TRPV3. The targeted deletion of FIH in mice and mice treated with activators of TRPV3 channels both display similar metabolic effects, including decreased adiposity and resistance to weight gain on a high fat diet [49,52]. So, the therapeutic inhibition of FIH in the context of obesity or diabetes may also result in beneficial outcomes through two independent pathways.

## 6. Conclusions

The recent identification of hydroxylation-mediated changes in TRPA1 and TRPV3 channel activity—although based on a limited number of highly focused studies—has expanded our understanding of the role of channel post-translational modifications, and the oxygen-dependent modulation of these channels. It has also uncovered new therapeutic strategies to modulate the activity of these specific channels by targeting the PHDs and FIH, while identifying potential TRP channel-mediated side effects as a consequence of targeting these hydroxylases to therapeutically regulate HIF activity.
